# A Two-Stage STAP Method Based on Fine Doppler Localization and Sparse Bayesian Learning in the Presence of Arbitrary Array Errors

**DOI:** 10.3390/s22010077

**Published:** 2021-12-23

**Authors:** Kun Liu, Tong Wang, Jianxin Wu, Jinming Chen

**Affiliations:** 1 National Lab of Radar Signal Processing, Xidian University, Xi’an 710071, China; kliu-1@stu.xidian.edu.cn (K.L.); jmchen1614@126.com (J.C.); 2 School of Electronics and Communication Engineering, Sun Yat-sen University, Guangzhou 510275, China; wujx65@mail.sysu.edu.cn

**Keywords:** airborne radar, arbitrary array error, clutter suppression, space-time adaptive processing, sparse Bayesian learning

## Abstract

In the presence of unknown array errors, sparse recovery based space-time adaptive processing (SR-STAP) methods usually directly use the ideal spatial steering vectors without array errors to construct the space-time dictionary; thus, the steering vector mismatch between the dictionary and clutter data will cause a severe performance degradation of SR-STAP methods. To solve this problem, in this paper, we propose a two-stage SR-STAP method for suppressing nonhomogeneous clutter in the presence of arbitrary array errors. In the first stage, utilizing the spatial-temporal coupling property of the ground clutter, a set of spatial steering vectors with array errors are well estimated by fine Doppler localization. In the second stage, firstly, in order to solve the model mismatch problem caused by array errors, we directly use these spatial steering vectors obtained in the first stage to construct the space-time dictionary, and then, the constructed dictionary and multiple measurement vectors sparse Bayesian learning (MSBL) algorithm are combined for space-time adaptive processing (STAP). The proposed SR-STAP method can exhibit superior clutter suppression performance and target detection performance in the presence of arbitrary array errors. Simulation results validate the effectiveness of the proposed method.

## 1. Introduction

Space-time adaptive processing (STAP) [1,2,3,4,5,6,7,8] is an effective approach for ground clutter suppression and low-velocity target detection in airborne radars. The performance of STAP mainly depends on the estimation accuracy of the clutter plus noise covariance matrix (CCM) of the cell under test (CUT). Generally, the independent and identically distributed (IID) target-free training samples adjacent to the CUT are used to estimate the CCM. According to the Reed–Mallett–Brennan (RMB) rule [9], to achieve an output signal-to-clutter-plus-noise ratio (SCNR) loss within 3 dB, the number of used IID training samples must be greater than twice the system degrees of freedom (DOFs). However, this requirement is hard to be satisfied in the practical heterogeneous and non-stationary clutter environment, thereby resulting in a severe performance degradation of the STAP algorithms.

Several low-sample methods have been developed to relieve the performance degradation caused by limited training data, such as reduced-dimension (RD) [10,11,12,13,14,15,16] algorithms, reduced-rank (RR) [17,18,19,20,21] algorithms, parametric adaptive matched filter (PAMF) algorithms [22,23], direct data domain (D3) [24,25] algorithms and knowledge-aided (KA) algorithms [26,27,28,29,30]. Although these algorithms can reduce the number of required training samples, they suffer from some drawbacks. The requirement of RR and RD algorithms is still hard to be satisfied, especially for large scale systems, the order for PAMF algorithms is hard to be determined, the system DOFs are significantly reduced for D3 algorithms and the exact prior knowledge of the environment is hard to obtain for KA algorithms.

Recently, with the development of sparse recovery (SR) techniques, sparse recovery based space-time adaptive processing (SR-STAP) methods have been extensively re-searched [31,32,33,34,35,36,37,38,39]. By utilizing the intrinsic sparsity of the clutter in angle-Doppler plane, SR-STAP recovers a signal with a sparse coefficient vector and a uniformly discretized space-time dictionary. Compared with the traditional STAP methods, SR-STAP can exhibit better clutter suppression performance in a very small training samples support. However, unfortunately, most SR algorithms, such as the iterative splitting and thresholding (IST) algorithm [40] and homotopy algorithm [41], need the fine tuning of one or more user parameters which affect the recovery results significantly. Sparse Bayesian learning (SBL) was proposed by Tipping and has been introduced to sparse signal recovery by Wipf for the single measurement vector (SMV) case and multiple measurement vector (MMV) case [42,43,44]. Different with the general SR algorithms, SBL is parameter-independent, which can guarantee the robustness of the algorithm in changing environment. Moreover, SBL can get favorable performance when the dictionary is highly coherent and its global minimum is always the sparsest solution. Thus, for its robustness and excellent performance, sparse Bayesian learning based space-time adaptive processing (SBL-STAP) [45,46] has received much attention.

However, SR-STAP methods rely on the accuracy of the sparse model and suffer performance degradation due to the model mismatch caused by array errors. Thus, several SR-STAP methods which can handle unknown array errors are developed. A sparsity-based STAP method considering array gain/phase error (AGPE-STAP) is proposed in [47], which combines a conventional sparsity-based STAP method and a conventional array gain/phase error calibration method. A sparsity-based STAP method with array gain/phase (GP) error self-calibration has been developed in [48], which iteratively solves an SR problem and an LS calibration problem. In [49], utilizing the specific structure of the mutual coupling matrix, a mutual coupling calibration method is developed for SBL-STAP by rearranging the received snapshots with the designed spatial-temporal selection matrix. In [50], under the framework of the alternating direction method (ADM), a constraint is added to the array GP errors, and the conventional sparsity-based STAP problem is transformed into a joint optimization problem of the angle-Doppler profile and the array GP errors. However, these SR-STAP methods are based on model errors and are only suitable for gain/phase calibration or mutual coupling calibration, in practice, various array errors often work together and some errors are difficult to model, in that case, these methods are no longer effective. Thus, an SR-STAP method which can handle the arbitrary array errors is urgently needed.

In this paper, we propose a two-stage SR-STAP method for suppressing nonhomo-geneous clutter in the presence of arbitrary array errors. In our two-stage SR-STAP method, the radar operates in two modes. In the first stage, radar operates in measurement mode, this mode needs a long coherent processing interval (CPI) to ensure sufficient Doppler resolution. Then, utilizing the spatial-temporal coupling property of the ground clutter, a set of spatial steering vectors with array errors are well estimated by fine Doppler localization. In the second stage, radar operates in STAP mode, in order to solve the model mismatch problem caused by array errors, we directly use these spatial steering vectors obtained in the first stage to construct the space-time dictionary, and then, the constructed dictionary and MSBL algorithm are combined for STAP. The main contributions of this paper are summarized as follows.

(1) A new two-stage SR-STAP method is proposed, in the presence of arbitrary array errors, the proposed two-stage SR-STAP method can obtain superior clutter suppression performance and target detection performance with limited training samples.

(2) Steering vector estimation for arbitrary array errors is developed, which is based on the spatial-temporal coupling property of the ground clutter. Relative to many existing array calibration methods which are only suitable for individual perturbation, the developed method can handle arbitrary array errors. Since it is free of the array model and based on clutter data, the developed method also avoids the model mismatch problem and has adaptability to the changing scenes.

(3) The developed method for estimating steering vectors is still effective when intrinsic clutter motion (ICM) is present, spatial steering vectors with array errors can also be well estimated when the pulse-to-pulse fluctuations are small.

The rest of the paper is organized as follows. In Section 2, the signal model with array errors is introduced. In Section 3, the proposed two-stage SR-STAP method is introduced. In Section 4, simulation results are provided to demonstrate the clutter suppression performance and target detection performance of the proposed method. Final conclusion is discussed in Section 5.

Notation: Boldface small letters denote vectors and boldface capital letters denote matrices. ${\left(\cdot \right)}^{T}$ and ${\left(\cdot \right)}^{H}$ represent the transpose and Hermitian transpose, respectively. R, $R^{+}$ and C represent the real filed, nonnegative real filed and complex filed, respec-tively. The expectation operator is represented by $E\left(\cdot \right)$. The symbols ⊗ and ⊙ denote the Kronecker product and Hadamard product, respectively. $diag\left(\cdot \right)$ represents a diagonal matrix with entries of the argument vector on the diagonal. The NK×NK identity matrix is defined as $I_{NK}$. ${∥\cdot ∥}_{F}$ denotes the Frobenius norm. ${∥\cdot ∥}_{2,0}^{}$ denotes a mixed norm defined as the number of non-zero elements of $l^{2}$-norms of the row vectors.

## 2. Signal Model

Consider an airborne pulsed Doppler radar system that employs a side-looking uniform linear array (ULA) consisting of *N* elements with an inter-element spacing *d* and *K* coherent pulses in a CPI at a constant pulse repetition frequency (PRF) $f_{PRF}$. Ignoring the influence of range ambiguity, the clutter plus noise echoes collected over all pulses, all elements and all range bins can be represented by

(1) $$Y=\left[y_{1},y_{2},\ldots ,y_{L}\right]$$

where $y_{l}$ is clutter plus noise data snapshot with array errors of the *l*th range bin, given by

(2) $$\begin{array}{cc} y_{l} & ={\left[y_{11l},y_{21l},\ldots ,y_{N1l},\ldots ,y_{1Kl},y_{2Kl},\ldots ,y_{NKl}\right]}^{T}\\ \; & =\sum_{i=1}^{N_{c}}\varsigma_{c,i}s_{c,i}+n_{l}\\ \; & =\sum_{i=1}^{N_{c}}\varsigma_{c,i}\left(b\left(f_{di}^{}\right)⊗a\left(f_{si}^{}\right)\right)+n_{l} \end{array}$$

where $N_{c}$ is the number of independent clutter sources, $\varsigma_{c,i}$ is the random complex amplitude, $s_{c,i}\;=\;b\left(f_{di}\right)⊗a\left(f_{si}\right)$ is the spatial-temporal steering vector with array errors of the *i*th clutter patch, $a\left(f_{si}\right)\;=\;G_{c,i}\bar{a}\left(f_{si}\right)$ is the spatial steering vector with array errors of the *i*th clutter patch, $G_{c,i}$ is the array error matrix of the *i*th clutter patch, $n_{l}$ is a Gaussian noise vector with zero mean and covariance matrix $\sigma^{2}I$, $\sigma^{2}$ is the noise power, I is the identity matrix, $b\left(f_{di}\right)$ and $\bar{a}\left(f_{si}\right)$ are the corresponding temporal steering vector and the ideal spatial steering vector without array errors, and

(3) $$b\left(f_{di}\right)\;=\;{\left[1,\exp\left(j2\pi f_{di}\right),\ldots ,\exp\left(j2\pi \left(K-1\right)f_{di}\right)\right]}^{T}$$

(4) $$\bar{a}\left(f_{si}\right)\;=\;{\left[1,\exp\left(j2\pi f_{si}\right),\ldots ,\exp\left(j2\pi \left(N-1\right)f_{si}\right)\right]}^{T}$$

where $f_{si}^{}=d\cos\phi_{i}/\lambda$ and $f_{di}^{}=2v_{p}\cos\phi_{i}/\left(\lambda f_{PRF}\right)$ are the normalized spatial fre-quency and the normalized Doppler frequency of the *i*th clutter patch, $\phi_{i}$ is the corre-sponding spatial cone angle, λ is the wavelength, $v_{p}$ is the velocity of the platform.

In practice, the gain and delay of each sensor are usually not identical due to different aging rates or imperfect manufacturing, which causes gain and phase errors. The errors can be represented by a N×N complex diagonal matrix $G_{gain}$ [4]

(5) $$G_{gain}=diag\left(\left[g_{1},g_{2},\cdots ,g_{N}\right]\right)$$

where $g_{n}\;=\;\left(1+\Delta \alpha_{n}\right)e^{j\Delta \varphi_{n}}$, $\Delta \alpha_{n}$ and $\Delta \varphi_{n}$ are the gain error and phase error of the *n*th sensor, respectively.

Due to closed distance, the interactions among sensors generate mutual coupling. The mutual coupling can be represented by the following N×N symmetric Toeplitz matrix $G_{mutual}$ [4]

(6) $$G_{mutual}=\left[\begin{array}{cccccc} 1 & c_{1} & \cdots & c_{q} & \cdots & 0\\ c_{1} & 1 & c_{1} & \cdots & \ddots & \vdots \\ \vdots & c_{1} & 1 & c_{1} & \cdots & c_{q}\\ c_{q} & \ddots & c_{1} & 1 & c_{1} & \vdots \\ \vdots & \vdots & \ddots & c_{1} & 1 & c_{1}\\ 0 & \cdots & c_{q} & \cdots & c_{1} & 1 \end{array}\right]$$

where $c_{i}\left(i=1,2,\ldots ,q\right)$ denotes the complex mutual coupling coefficient, q⩽N, which means that the mutual coupling can be ignored when the element spacing is greater than *q* inter-element spacing.

In order to obtain a certain geometry of array, each sensor must be in the precise location. However, in practice, this requirement is sometimes difficult to satisfy, which causes the sensor location errors. The error vector of the *i*th clutter patch caused by sensor location errors can be written as [4]

(7) $$e_{pi}={\left[1,e^{j2\pi \Delta_{1}\cos\phi_{i}/\lambda },\cdots ,e^{j2\pi \Delta_{N-1}\cos\phi_{i}/\lambda }\right]}^{T}$$

where $\Delta_{0}\;=\;0$, $\Delta_{j}\left(j=1,2,\ldots ,N-1\right)$ are the random numbers represent the location errors for each sensor.

Let $G_{othersi}\in C^{N\times N}$ denotes other array perturbations encountered at the *i*th clutter patch, the array error matrix $G_{c,i}$ can be formulated as

(8) $$G_{c,i}\;=\;G_{gain}G_{mutual}diag\left(e_{pi}\right)G_{othersi}$$

## 3. Proposed Method

In this section, we propose a two-stage SR-STAP method for suppressing nonhomo-geneous clutter in the presence of arbitrary array errors.

### 3.1. Steering Vector Estimation

In the first stage, radar operates in measurement mode, assuming that the number of pulses in a CPI is $K_{1}$, to promise sufficient Doppler resolution, $K_{1}$ should be a large value. From (2), we get the clutter plus noise data snapshot of the *l*th range bin.

(9) $$y_{l}=\sum_{i=1}^{N_{c}}\varsigma_{c,i}\left(b\left(f_{di}^{}\right)⊗a\left(f_{si}^{}\right)\right)+n_{l}$$

Without consideration of the ICM, the relationship of spatial frequency $f_{si}^{}$ and temporal frequency $f_{di}^{}$ is represented by

(10) $$f_{si}^{}\;=\;\frac{df_{PRF}}{2v_{p}}f_{di}^{}\;,\;\;i=1,2,\cdots ,N_{c}$$

It means that clutter patches can be localized either by a spatial filter or by a Doppler filter. Generally, the number of pulses in a CPI is larger than the number of elements in the array, so, it is easier to create a narrow Doppler filter. Moreover, the ultra-low sidelobe of a Doppler filter is more reasonable than that of a spatial filter. Thus, clutter localization is preferred to be realized by fine Doppler localization. The *k*th Doppler filter output is given by

(11) $$\begin{array}{cc} X_{kl}\; & =T_{k}^{H}y_{l}={\left(f_{k}⊗I_{N}\right)}^{H}y_{l}\\ \; & =\sum_{i=1}^{N_{c}}\varsigma_{c,i}{\left(f_{k}⊗I_{N}\right)}^{H}\left(b\left(f_{di}^{}\right)⊗a\left(f_{si}^{}\right)\right)+{\left(f_{k}⊗I_{N}\right)}^{H}n_{l}\\ \; & =\sum_{i=1}^{N_{c}}\varsigma_{c,i}\left(f_{k}^{H}b\left(f_{di}^{}\right)\right)⊗\left(I_{N}a\left(f_{si}^{}\right)\right)+{\left(f_{k}⊗I_{N}\right)}^{H}n_{l}\\ \; & =\sum_{i=1}^{N_{c}}\varsigma_{c,i}\left(f_{k}^{H}b\left(f_{di}^{}\right)\right)a\left(f_{si}^{}\right)+{\tilde{n}}_{l} \end{array}$$

where $T_{k}=\left(f_{k}⊗I_{N}\right)$ is the transformation matrix, $f_{k}\;=\;t_{f}⊙u_{k}$ is the Doppler filter coefficient vector of the *k*th Doppler filter, $t_{f}$ is a ultra-low sidelobe taper, $u_{k}=[1,\exp\left(j2\pi k/K_{1}\right),\cdots ,$$\exp\left(j2\pi k\left(K_{1}-1\right)/K_{1}\right){]}^{T}$, ${\tilde{n}}_{l}\;=\;{\left(u_{k}⊗I_{N}\right)}^{H}n_{l}$ is the additive Gaussian noise, and

(12) $$pbr\left(f_{dk}^{}-f_{di}^{}\right)\;=\;f_{k}^{H}b\left(f_{di}^{}\right)$$

is the low-pass filter response with the passband of

(13) $$\left|f_{di}^{}-f_{dk}^{}\right|⩽\frac{D_{w}}{2}$$

where $f_{dk}^{}$ is the center frequency of the *k*th Doppler filter, $D_{w}$ is the Doppler frequency passband width (DFPW). Then, (11) can be recast as

(14) $$X_{kl}\;=\;\sum_{i=1}^{N_{c}}\varsigma_{c,i}pbr\left(f_{dk}^{}-f_{di}^{}\right)a\left(f_{si}^{}\right)+{\tilde{n}}_{l}$$

According to the Doppler frequency passband of the *k*th Doppler filter, we can get the associated spatial frequency passband of the clutter component by substituting (10) into (13)

(15) $$\frac{df_{PRF}}{2v_{p}}f_{dk}^{}-\frac{df_{PRF}}{4v_{p}}D_{w}⩽f_{si}^{}⩽\frac{df_{PRF}}{2v_{p}}f_{dk}^{}+\frac{df_{PRF}}{4v_{p}}D_{w}$$

The width of the spatial frequency passband is

(16) $$\Delta \;=\;\frac{df_{PRF}}{2v_{p}}D_{w}$$

For a Doppler filter with ultra-low sidelobes, the gain of the stopband is negligible relative to the passband. Without consideration of the components in the stopband of the Doppler filter, (14) can be written as

(17) $$X_{kl}\;=\;\sum_{i=N_{pk}}^{N_{qk}}\xi_{c,i}a\left(f_{si}^{}\right)+{\tilde{n}}_{l}$$

where $\xi_{c,i}\;\;=\;\;\varsigma_{c,i}pbr\left(f_{dk}^{}-f_{di}^{}\right)$, $N_{pk}$ and $N_{qk}$ are the bounded indexes of the spatial frequency passband corresponding to the *k*th Doppler filter.

Similar to the Doppler beam sharpening (DBS) radar, we define a sharpening ratio as

(18) $$\kappa \;=\;\frac{\theta_{\mathrm{mainlobe}}}{\Delta }=\frac{2v_{p}}{Ndf_{PRF}D_{w}}$$

where $\theta_{\mathrm{mainlobe}}$ is the mainlobe beamwidth.

For an untapered Doppler filter, the distance between its two first nulls is $2/K_{1}$, which is larger than its DFPW. Therefore, when $K_{1}$ is large, a narrow Doppler filter with a small DFPW can be obtained. However, its sidelobe level is high (the first sidelobe is at −13.4 dB); thus, the sidelobe gain of an untapered Doppler filter cannot be ignored. A heavy tapered Doppler filter can obtain ultra-low sidelobes, but the obtainment is at the cost of a broadening mainlobe, and thereby resulting a larger DFPW. We define the DFPW to be the width of the Doppler frequency range where the drop of the power gain of a Doppler filter is less than 40 dB. For a Doppler filter with ultra-low sidelobes, the power gain is negligible outside this range. It is difficult to get the analytical DFPW of a tapered Doppler filter, but we can give a reasonable value based on our experience. For example, when a Chebyshev taper with sidelobe level of −80 dB is used, by experience, we know that $5/K_{1}$ is a reasonable DFPW value, i.e., $D_{w}\;=\;5/K_{1}$. Substituting $D_{w}\;=\;5/K_{1}$ into (16), we get the spatial frequency passband width corresponding to the DFPW of a Doppler filter with a 80 dB Chebyshev taper.

(19) $$\Delta =\frac{5df_{PRF}}{2v_{p}K_{1}}$$

Substituting (19) into (18) yields

(20) $$\kappa \;=\;\frac{\theta_{\mathrm{mainlobe}}}{\Delta }\;=\;\frac{2v_{p}K_{1}}{5Ndf_{PRF}}$$

Define the correlation coefficient of $a\left(f_{si}^{}\right)$ and $a\left(f_{si}\;+\;\Delta /2\right)$ as

(21) $$cc=\frac{\left|a^{H}\left(f_{si}\;+\;\frac{\Delta }{2}\right)a\left(f_{si}\right)\right|}{\sqrt{a^{H}\left(f_{si}\;+\;\frac{\Delta }{2}\right)a\left(f_{si}\;+\;\frac{\Delta }{2}\right)}\sqrt{a^{H}\left(f_{si}\right)a\left(f_{si}\right)}}$$

**Figure 1:**
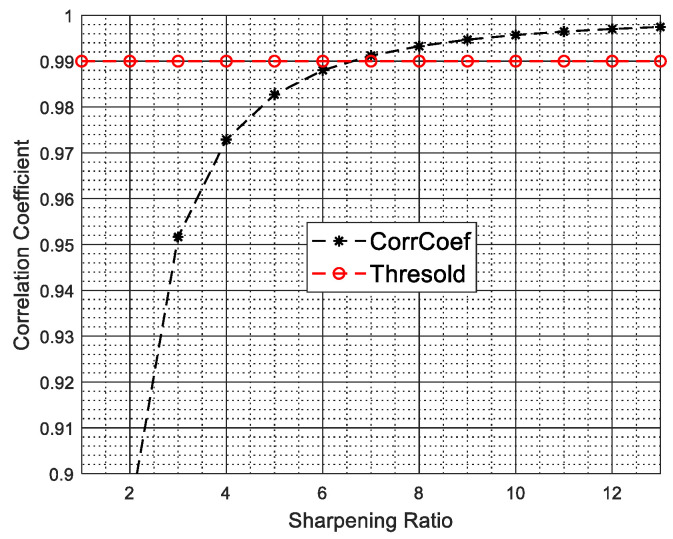
Correlation coefficient of $a\left(f_{si}^{}\right)$ and $a\left(f_{si}\;+\;\Delta /2\right)$ versus the sharpening ratio.

Thus, as the number of pulses $K_{1}$ increases, the sharpening ratio κ becomes larger and Δ becomes smaller, as a result, the correlation coefficient of $a\left(f_{si}^{}\right)$ and $a\left(f_{si}\;+\;\Delta /2\right)$ becomes larger. Figure 1 depicts the correlation coefficient of $a\left(f_{si}^{}\right)$ and $a\left(f_{si}\;+\;\Delta /2\right)$ versus the sharpening ratio κ, where $v_{p}=150\;m/s$, N=8, d=0.15m, $f_{PRF}=2000\;\mathrm{Hz}$ and $K_{1}$ changes from 40 to 520 at intervals of 40.

In Figure 1, the dotted line with symbol * shows the correlation coefficient of $a\left(f_{si}^{}\right)$ and $a\left(f_{si}^{}+\Delta /2\right)$ versus the sharpening ratio κ and the dotted line with symbol ∘ denotes a threshold value. From Figure 1, we can observe that when the sharpening ratio κ is larger than 6.4, the correlation coefficient of $a\left(f_{si}^{}\right)$ and $a\left(f_{si}^{}+\Delta /2\right)$ is greater than 0.99, i.e., in this case, if κ is larger than 6.4 (the number of pulses in a CPI is larger than 256), $a\left(f_{si}^{}+\Delta /2\right)$ can be well approximated by $a\left(f_{si}^{}\right)$.

When the sharpening ratio κ is large, $a\left(f_{sk}^{}\pm \Delta /2\right)\approx a\left(f_{sk}^{}\right)$, (17) can be simplified as

(22) $$X_{kl}\;=\;\mu_{kl}a\left(f_{sk}^{}\right)+{\tilde{n}}_{l}$$

where $\mu_{kl}\;=\;\sum_{i=N_{pk}}^{N_{qk}}\xi_{c,i}$, $f_{sk}^{}$ is the normalized spatial frequency corresponding to the center Doppler frequency of the *k*th Doppler filter, $a\left(f_{sk}^{}\right)$ is the corresponding spatial steering vector.

To alleviate the bad influence of ${\tilde{n}}_{l}$, multiple range gates are utilized to estimate $a\left(f_{sk}^{}\right)$, according to (22), the covariance matrix of $X_{kl}$ can be written as

(23) $$R_{kl}\;=\;E\left(X_{kl}X_{kl}^{H}\right)\;=\;\gamma_{kl}^{2}a\left(f_{sk}^{}\right)a^{H}\left(f_{sk}^{}\right)\;+\;{\tilde{\sigma }}^{2}I_{N}$$

where $\gamma_{kl}^{2}\;=\;E\left({\left|\mu_{kl}\right|}^{2}\right)$, $E\left({\tilde{n}}_{l}{\tilde{n}}_{l}^{H}\right)={\tilde{\sigma }}^{2}I_{N}$, ${\tilde{\sigma }}^{2}$ is the additive noise power. In practice, $R_{kl}$ is unknown and can be substituted by the sample covariance matrix, i.e.,

(24) $${\hat{R}}_{kl}\;=\;\frac{1}{L}\sum_{l=1}^{L}X_{kl}X_{kl}^{H}$$

Under the high clutter-to-noise ratio (CNR) case, $\gamma_{kl}^{2}/{\tilde{\sigma }}^{2}\gg 1$ and it is valid to say that the number of large eigenvalues of ${\hat{R}}_{kl}$ is 1. Thus, we can perform singular value decomposition (SVD) on ${\hat{R}}_{kl}$ and $a\left(f_{sk}^{}\right)$ is estimated by the eigenvector associated with the largest eigenvalue.

When ICM is present, the pulse-to-pulse fluctuations will cause a broadening of the Doppler spectrum of a single clutter return and the relation in (10) does not hold. In this case, for a single clutter echo, its Doppler frequency range can be written as

(25) $$f_{di}-\frac{D_{b}}{2}⩽f_{d}⩽f_{di}^{}+\frac{D_{b}}{2}$$

where Db=2σv2σvλfPRFλfPRF is the width of the Doppler spectrum, $\sigma_{v}$ is the velocity standard deviation caused by ICM [1].

By substituting (10) into (25), we get the associated spatial frequency range

(26) $$\frac{df_{PRF}}{2v_{p}}f_{di}^{}-\frac{df_{PRF}}{4v_{p}}D_{b}⩽f_{s}⩽\frac{df_{PRF}}{2v_{p}}f_{di}^{}+\frac{df_{PRF}}{4v_{p}}D_{b}$$

And the width of the spatial frequency range is

(27) $$\Delta_{b}\;=\;\frac{df_{PRF}}{2v_{p}}D_{b}$$

When the Doppler spectrum broadening caused by ICM is much smaller than the DFPW of the heavy tapered Doppler filter, i.e., $D_{b}\ll D_{w}$, the inequality $\Delta_{b}\ll \Delta$ holds. As a result, the correlation coefficient of $a\left(f_{si}^{}\right)$ and $a\left(f_{si}+\Delta_{b}/2\right)$ is approximately 1 when the sharpening ratio κ is large, and in this case, we can say that the Doppler frequency range given in (25) corrsponds to a single spatial frequency $f_{si}^{}$. Thus, when the velocity standard deviation $\sigma_{v}$ caused by ICM is small, the broadening of the Doppler spectrum has little effect on estimating the spatial steering vectors and the proposed method for estimating spatial steering vector still works well.

**Figure 2:**
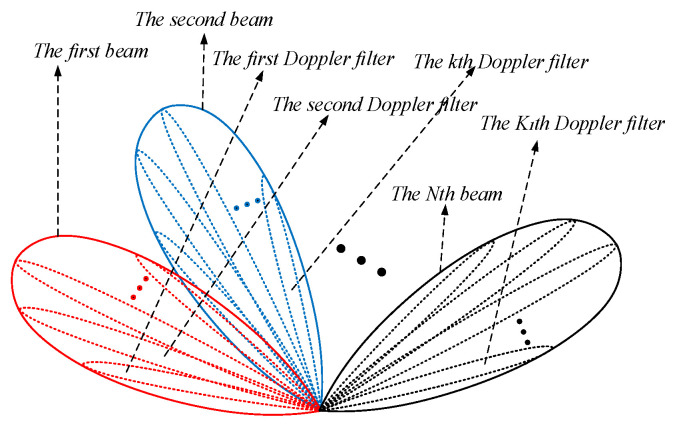
Beam scanning and fine Doppler localization.

In practice, clutter from the sidelobes and the nulls of the array pattern is much weaker than that from the mainlobe. Besides, the reflection coefficients are small in some unknown clutter areas. In addition, the adjacent range gates used to estimate $R_{kl}$ may include strong moving targets and other unwanted components. In these cases, the estimation accuracy of $R_{kl}$ or the condition $\gamma_{kl}^{2}/{\tilde{\sigma }}^{2}\gg 1$ cannot be well guaranteed, which causes an inaccurate estimate of $a\left(f_{sk}\right)$. Thus, beam scanning and secondary data selection are necessary. Figure 2 describes the process of beam scanning and fine Doppler localization. Firstly, to guarantee the gain of the array in all clutter regions, multiple beams, such as a group of *N* orthogonal Fourier beams, are used to cover all the azimuth angles; thus, we can get the ground clutter data of all range gates under each spatial beam. Then, for the reason that the angle resolution in the spatial domain is low while the Doppler resolution in the temporal domain is high, a group of $K_{1}$ Doppler filters are used for better localization of the ground clutter. Thus, we can obtain the output data of all range gates under each heavy tapered Doppler filter by the fine Doppler localization of the ground clutter data. Each spatial beam will cover several Doppler filters and *N* spatial beams will cover all $K_{1}$ Doppler filters, and the gain of the array in these clutter areas corresponding to the DFPW of each Doppler filter can be well guaranteed. Thus, by processing the output data of each heavy tapered Doppler filter in turn, a set of $K_{1}$ spatial steering vectors can be well estimated.

(28) $$\varepsilon_{l}\;=\;{\left|{\hat{a}}_{0}^{H}\left(f_{sk}^{}\right)X_{kl}\right|}^{2}$$

and an angle selection parameter $\rho_{l}$

(29) $$\rho_{l}=\frac{\left|{\hat{a}}_{0}^{H}\left(f_{sk}^{}\right)X_{kl}\right|}{\sqrt{{\hat{a}}_{0}^{H}\left(f_{sk}^{}\right){\hat{a}}_{0}^{}\left(f_{sk}^{}\right)}\sqrt{{X_{kl}}^{H}X_{kl}}}$$

where ${\hat{a}}_{0}^{}\left(f_{sk}^{}\right)$ is the initial estimated spatial steering vector by utilizing all range gates. According to the definition of $\varepsilon_{l}$ and $\rho_{l}$ given in (28) and (29), we find that $\varepsilon_{l}$ is dependent on both the direction and amplitude of $X_{kl}$ and $\rho_{l}$ is only dependent on the direction of $X_{kl}$. Thus, firstly, we use a power selection parameter $\varepsilon_{l}$ to pick out the range gates which may be strong clutter or strong outliers. Then, we use an angle selection parameter $\rho_{l}$ to kick out the possible outliers, such as strong moving targets or strong interference, whose directions are different from $X_{kl}$. Thereafter, these range gates which may be strong clutter can be preserved and the possible outliers can be removed.

**Figure 3:**
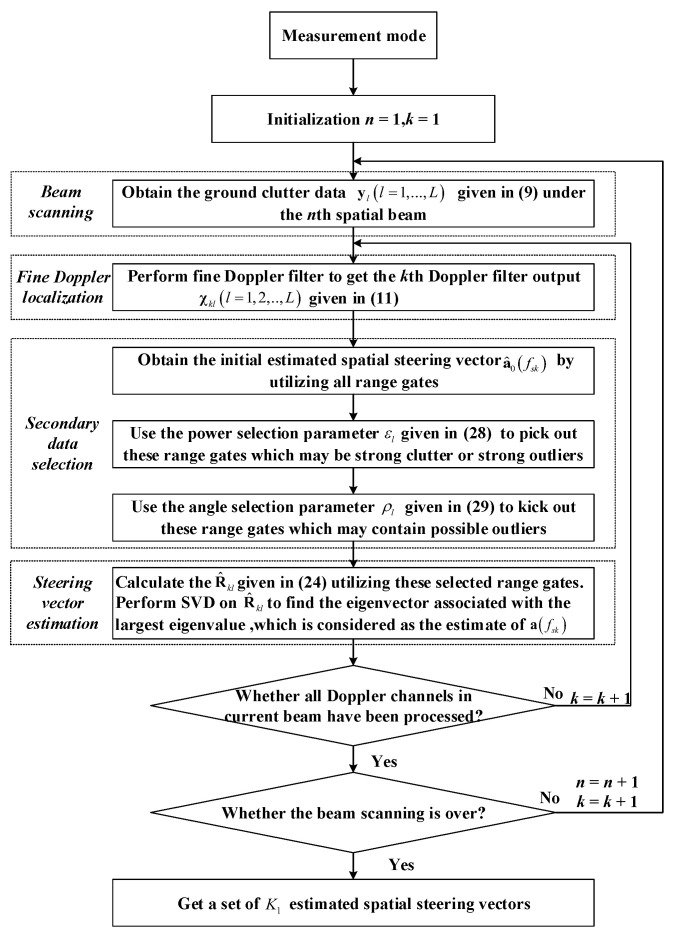
The flow chart of the first stage of the proposed two-stage SR-STAP method.

Figure 3 plots the flow chart of the first stage of the proposed two-stage SR-STAP method. In the first stage of our two-stage SR-STAP method, our goal is to estimate a set of spatial steering vectors with array errors. Firstly, in the beam scanning step, we can get the ground clutter data of all range gates given in (9) under the first spatial beam. Then, in the fine Doppler localization step, we can obtain the output data of all range gates given in (11) under the first heavy tapered Doppler filter by the fine Doppler localization of the ground clutter data. Then, for the secondary data selection step, we firstly obtain the initial estimated spatial steering vector by utilizing the output data of all range gates given in (11); then we use the power selection parameter $\varepsilon_{l}$ given in (28) to pick out these range gates which may be strong clutter or strong outliers; finally, we use the angle selection parameter $\rho_{l}$ given in (29) to kick out these range gates which may contain possible outliers. Next, for the steering vector estimation step, we calculate the ${\hat{R}}_{kl}$ by (24) utilizing these selected range gates and perform SVD on ${\hat{R}}_{kl}$ to find the eigenvector $\hat{a}\left(f_{sk}\right)$ associated with the largest eigenvalue, which is considered as the estimate of $a\left(f_{sk}\right)$. Here we can get the spatial steering vector with array errors corresponding to the first heavy tapered Doppler filter. Then, we need to judge whether all the Doppler channel contained in the current beam have been processed. If it has not been finished, we should assume k=k+1 and back to the beam scanning step. If the answer is Yes, we need to judge whether the beam scanning has been finished and if has not, we should assume n=n+1 and back to the fine Doppler localization step. When the beam scanning ends and all $K_{1}$ Doppler bins are processed, a set of $K_{1}$ spatial steering vectors with array errors are well estimated by fine Doppler localization.

The procedures of the first stage of the proposed method are summarized as follows:

Step 1: Obtain the initial estimated spatial steering vector ${\hat{a}}_{0}^{}\left(f_{sk}^{}\right)$ corresponding to the *k*th Doppler filter.

Step 2: Compute the values of power selection parameter $\varepsilon_{l}$ and angle selection parameter $\rho_{l}$ for all range gates according to (28) and (29).

Step 3: Find *p* range gates corresponding to *p* maximal values of $\varepsilon_{l}$ among all *L* range gates, i.e., $\left\{l_{1},l_{2},\cdots ,l_{p}\right\}=\underset{l}{\arg\max}\left\{\varepsilon_{l}\right\},l=1,2,\ldots ,L$.

Step 4: Find *q* range gates corresponding to *q* maximal values of $\rho_{l}$ among all *p* range gates selected in step 3, i.e., $\left\{{\dot{l}}_{1},{\dot{l}}_{2},\cdots ,{\dot{l}}_{q}\right\}=\underset{l}{\arg\max}\left\{\rho_{l}\right\},l=l_{1},l_{2},\cdots ,l_{p}$.

Step 5: Calculate the ${\hat{R}}_{kl}$ given in (24) utilizing *q* range gates selected in step 4. Perform SVD on ${\hat{R}}_{kl}$ to find the eigenvector $\hat{a}\left(f_{sk}\right)$ associated with the largest eigenvalue, which is considered as the estimate of $a\left(f_{sk}\right)$.

Step 6: Go back to step 1 until the beam scanning ends and all $K_{1}$ Doppler bins are processed.

### 3.2. SR-STAP Method

In the second stage of our two-stage SR-STAP method, we firstly use these spatial steering vectors obtained in the first stage to construct the space-time dictionary, and then, since the MSBL algorithm has been demonstrated a robust, sparse enough, parameter-independent algorithm in the presence of noise, the existing multiple measurement vector sparse Bayesian learning based space-time adaptive processing (MSBL-STAP) [45] method is adopted.

In the second stage, radar operates in STAP mode, since we have already measured a set of spatial steering vectors with array errors in the first stage; thus, in this mode, high Doppler resolution is not needed, assuming that the pulse number in a CPI is $K_{2}$, in general, $K_{2}<K_{1}$. To solve the model mismatch problem caused by array errors, we need to select $N_{s}$ spatial steering vectors from the $K_{1}$ spatial steering vectors obtained in the first stage and use these selected steering vectors to construct the space-time dictionary. Then, the received data snapshot of *L* range bins can be expressed by

(30) Y=DΨ+N

where $\Psi =\left[\beta^{\left(1\right)},\beta^{\left(2\right)},\cdots ,\beta^{\left(L\right)}\right]\in R^{N_{s}N_{d}\times L}$ is the solution matrix with each row representing a possible clutter source, $N=\left[n^{\left(1\right)},n^{\left(2\right)},\cdots ,n^{\left(L\right)}\right]\in C^{NK\times L}$ is a noise matrix whose entries are Gaussian with zero mean and variance $\sigma^{2}$, $D=\left[s_{1},s_{2},\ldots ,s_{M}\right]$ is the space-time dictionary, $M=N_{s}N_{d}$ is the number of the grid points of the whole angle-Doppler plane, $N_{s}\;=\;\rho_{s}N\leq K_{1}\left(\rho_{s}>1\right)$ is the number of angle bins, $N_{d}\;=\;\rho_{d}K_{2}\left(\rho_{d}>1\right)$ is the number of Doppler bins, $s_{m}=b\left(f_{dm}\right)⊗\hat{a}\left(f_{sm}\right)$ is the spatial-temporal steering vector with array errors of the *m*th grid point, $\hat{a}\left(f_{sm}\right)$ is the estimated spatial steering vector with array errors of the *m*th grid point, $b\left(f_{dm}\right)$ is the temporal steering vector of the *m*th grid point.

The angle-Doppler profile Ψ is obtained by solving the following optimization problem

(31) $$\min_{\Psi }{∥Y-D\Psi ∥}_{F}^{2}\;\;\;s.t.{∥\Psi ∥}_{2,0}^{}\leq r_{s}$$

where $r_{s}\in R^{+}$ is the degrees of the clutter sparsity (DOSs). A convex relaxation of (31) is

(32) $$\min_{\;\lambda ,\Psi }\;{∥Y-D\Psi ∥}_{F}^{2}+\sum_{m=1}^{M}\;\lambda {∥\Psi_{i\cdot }∥}_{2}$$

From a Bayesian perspective, (32) is equivalent to maximum a posterior probability (MAP) with the prior probability density function (PDF) $p∼\exp\left(-\sum_{i=1}^{M}{∥\Psi_{i\cdot }∥}_{2}\right)$ [43]. According to the measurement model in (30), we get the Gaussian likelihood function

(33) $$p\left(Y|\Psi ;\sigma^{2}\right)={\left(\pi \sigma^{2}\right)}^{-NKL}\exp\left(-\sigma^{-2}{∥Y-D\Psi ∥}_{F}^{2}\right)$$

Assuming that each column in Ψ obeys a complex Gaussian prior

(34) $$\beta^{\left(l\right)}∼N\left(0,\Gamma \right)$$

where 0 is a zero vector, Γ=diag(ζ), $\zeta \;=\;\left[\zeta_{1},\zeta_{2},\ldots ,\zeta_{M}\right]$ are the hyperparameters con-trolling the prior covariance of $\beta_{}^{\left(l\right)}$ and its values can be viewed as the power of the clutter sources. Then the prior PDF of Ψ can be represented as

(35) $$p\left(\Psi ;\Gamma \right)=\pi^{-ML}\Gamma^{-L}\exp\left(-\sum_{l=1}^{L}\beta^{{\left(l\right)}^{H}}\Gamma^{-1}\beta^{\left(l\right)}\right)$$

Combining the prior and likelihood, we get the posterior PDF of Ψ

(36) $$p\left(\Psi |Y;\Gamma ,\sigma^{2}\right)=\frac{p\left(Y|\Psi ;\sigma^{2}\right)p\left(\Psi ;\Gamma \right)}{\int p\left(Y|\Psi ;\sigma^{2}\right)p\left(\Psi ;\Gamma \right)d\Psi }$$

Actually, the sparsity profile Ψ is estimated by the posterior mean μ, whose value is modulated by the hyperparameter vector ζ and $\sigma^{2}$. Thus, the task to estimate Ψ is shifted to estimate the hyperparameter vector ζ and $\sigma^{2}$. The latter can be effectively accomplished by an expectation maximization (EM) algorithm. The procedures of the EM algorithm are described as follows.

E step: According to (33) and (35), the joint PDF of $\left(Y,\Psi \right)$ at j+1 step is given by

(37) $$\begin{array}{cc} p\left(Y,\Psi_{j+1};\Gamma_{j},\sigma_{j}^{2}\right)\; & =p\left(Y|\Psi_{j+1};\sigma_{j}^{2}\right)p\left(\Psi_{j+1};\Gamma_{j}\right)\\ \; & =\pi^{-\left(NK+M\right)L}\Gamma_{j}^{-L}\sigma_{j}^{-2NKL}\\ \; & \cdot \exp\left\{\sum_{l=1}^{L}\left[-\sigma_{j}^{-2}{\left(y_{l}-{D\beta }_{j+1}^{{}^{\left(l\right)}}\right)}^{H}\left(y_{l}-{D\beta }_{j+1}^{{}^{\left(l\right)}}\right)-\beta {{}_{j+1}^{{}^{\left(l\right)}}}^{H}\Gamma_{j}^{-1}\beta_{j+1}^{{}^{\left(l\right)}}\right]\right\} \end{array}$$

Then, the marginal PDF of Y at j+1 step is represented as

(38) $$\begin{array}{cc} p\left(Y;\Gamma_{j},\sigma_{j}^{2}\right)\; & =\int p\left(Y,\Psi_{j+1};\Gamma_{j},\sigma_{j}^{2}\right)d\Psi_{j+1}\\ \; & =\pi^{-NKL}{\left|\sigma_{j}^{2}I+D\Gamma_{j}D^{H}\right|}^{-L}\\ \; & \cdot \exp\left(\sum_{l=1}^{L}y_{l}^{H}{\left(\sigma_{j}^{2}I+D\Gamma_{j}D^{H}\right)}^{-1}y_{l}\right) \end{array}$$

By combining (37) and (38), we get the posterior PDF of Ψ at j+1 step

(39) $$\begin{array}{cc} p\left(\Psi_{j\;+\;1}|Y;\Gamma_{j},\sigma_{j}^{2}\right)\; & =\frac{p\left(Y,\Psi_{j+1};\Gamma_{j},\sigma_{j}^{2}\right)}{p\left(Y;\Gamma_{j},\sigma_{j}^{2}\right)}\\ \; & =\pi^{-ML}{\left|\Sigma_{j+1}\right|}^{-L}\\ \; & .\exp\left[\sum_{l=1}^{L}-{\left(\beta_{j+1}^{{}^{\left(l\right)}}-\mu_{j+1}^{{}^{\left(l\right)}}\right)}^{H}\Sigma_{j+1}^{-1}\left(\beta_{j+1}^{{}^{\left(l\right)}}-\mu_{j+1}^{{}^{\left(l\right)}}\right)\right] \end{array}$$

where $\mu_{j+1}^{{}^{}}$ is the mean matrix and $\Sigma_{j+1}$ is the covariance matrix, given by

(40) $$\mu_{j+1}^{{}^{}}\;=\;\Gamma_{j}D^{H}{\left(\sigma_{j}^{2}I+D\Gamma_{j}D^{H}\right)}^{-1}Y$$

(41) $$\Sigma_{j+1}\;=\;\Gamma_{j}-\Gamma_{j}D^{H}{\left(\sigma_{j}^{2}I+D\Gamma_{j}D^{H}\right)}^{-1}D\Gamma_{j}$$

M step: At M-step, we estimate $\zeta_{j+1}$ and $\sigma_{j+1}^{2}$ by using a Type-II maximum likelihood [42], i.e.,

(42) $$\left[\zeta_{j+1},\sigma_{j\;+\;1}^{2}\right]=\underset{\Gamma ,\sigma^{2}}{\arg\max}\left[E\left(\ln p\left(Y,\Psi_{j+1};\Gamma_{j},\sigma_{j}^{2}\right)\right)\right]$$

Because of decoupling [43], (42) can be divided into two optimization problems

(43) $$\zeta_{j+1}\;=\;\underset{\Gamma }{\arg\max}\left[E\left(\ln\left(p\left(\Psi_{j+1};\Gamma_{j}\right)\right)\right)\right]$$

(44) $$\sigma_{j+1}^{2}\;=\;\underset{\sigma^{2}}{\arg\max}\left[E\left(\ln\left(p\left(Y|\Psi_{j+1};\sigma_{j}^{2}\right)\right)\right)\right]$$

Substituting (35) into (43) yields

(45) $$\zeta_{m,j+1}=\frac{\sum_{l=1}^{L}\mu {{}_{m,j+1}^{\left(l\right)}}^{2}}{L}+\Sigma_{m,j+1}$$

where $\mu_{m,j+1}^{\left(l\right)}$ is the *m*th component of $\mu_{j+1}^{\left(l\right)}$, $\Sigma_{m,j+1}$ is the *m*th component of the main diagonal of $\Sigma_{j+1}$.

Substituting (33) into (44) yields

(46) $$\sigma_{j+1}^{2}\;=\;\frac{\left(1/L\right){∥Y-{D\mu }_{j+1}^{{}^{}}∥}_{F}^{2}+\sigma_{j}^{2}\sum_{m=1}^{M}\left(1-\left(\Sigma_{m,j+1}/\zeta_{m,j}\right)\right)}{NK}$$

The iteration for updating ζ and $\sigma^{2}$ ends when a predetermined criteria is satisfied. Such as, $∥\zeta_{j+1}-\zeta_{j}∥/∥\zeta_{j}∥⩽\delta$, where δ is a small enough positive threshold. Then, the CCM can be calculated by

(47) $$R_{c+n}=\frac{1}{L}\sum_{l=1}^{L}{\sum_{m=1}^{M}\left|\beta_{m}^{\left(l\right)}\right|}^{2}s_{m}s_{m}^{H}+\alpha \sigma^{2}I_{NK}$$

where α is a real constant. Based on the minimum variance distortionless response (MVDR) principle, we get the optimal STAP weight vector

(48) $$w=\frac{R_{c+n}^{-1}s_{t}}{s_{t}^{H}R_{c+n}^{-1}s_{t}}$$

where $s_{t}=b\left(f_{dt}\right)⊗\bar{a}\left(f_{st}\right)$ is the target spatial-temporal steering vector with the normalized Doppler frequency of $f_{dt}$ and the normalized spatial frequency of $f_{st}$.

The procedures of the second stage of the proposed method are summarized as follows:

Step 1: Construct the dictionary D using these spatial steering vectors obtained in the first stage, give the initial values $\zeta_{0}\;=\;1$, $\sigma_{0}^{2}\;=\;0.1$.

Step 2: Compute the mean matrix $\mu_{j+1}^{{}^{}}$ and the covariance matrix $\Sigma_{j+1}$ using (40) and (41).

Step 3: Update $\zeta_{j+1}$ and $\sigma_{j+1}^{2}$ using (45) and (46).

Step 4: Continue step 2 and step 3 until the predetermined criteria is satisfied.

Step 5: Calculate the CCM $R_{c+n}$ using (47), where Ψ≈μ.

Step 6: Compute the optimal STAP weight w using (48).

## 4. Numerical Experiments

In this section, numerical experiments are conducted to assess the performance of proposed method. The radar system parameters are given in Table 1. In the first stage, a Chebyshev taper with sidelobe level of −80 dB is used and the sharpening ratio κ is equal to 6.4. In the second stage, the discretized grids are set to be $N_{s}=32$ and $N_{d}=32$, i.e., $\rho_{s}=\rho_{d}\;=\;4$, the number of used training samples and the iteration termination threshold of MSBL-STAP algorithm are set to be 10 and δ=0.001, respectively. We use the signal to interference plus noise ratio (SINR) loss as a measure of clutter suppression performance, which is calculated by the ratio of output SINR and the signal to noise ratio (SNR) obtained by a match filter in a noise-only environment, i.e.,

(49) $$L_{SINR}=\frac{\sigma^{2}}{NK}\frac{{\left|w^{H}s_{t}\right|}^{2}}{w^{H}\mathrm{Rw}}$$

where w is the STAP weight vector, R is the known CCM. We also evaluate the target detection performance by the probability of detection (PD) versus SNR curves, which are achieved by utilizing the adaptive matched filter (AMF) detector [51], and the probability of false alarm rate (PFA) is set as $10^{-3}$, the target is assumed in the main beam direction with the normalized Doppler frequency 0.1, the threshold and probability of detection estimates are based on $10^{4}$ samples. Besides, all the simulation results of SINR loss are acquired through 100 Monte Carlo runs and all the PD to SNR curves are averaged over 1000 Monte Carlo trials.

To demonstrate the performance of proposed two-stage SR-STAP method in the presence of array errors, each perturbation is first considered separately, and then, their combined effects are demonstrated, finally, we also measure the effect of the presence of ICM on our two-stage SR-STAP method. We consider four cases in the simulation, (1) use the true spatial steering vectors with array errors to construct the space-time dictionary and perform MSBL-STAP, which is called TSV-MSBL, (2) use the estimated spatial steering vectors which are obtained by utilizing single range gate to construct the space-time dictionary and perform MSBL-STAP, which is called SESV-MSBL, (3) use the estimated spatial steering vectors which are obtained by utilizing multiple range gates to construct the space-time dictionary and perform MSBL-STAP, which is called MESV-MSBL, (4) use the ideal spatial steering vectors without array errors to construct the space-time dictionary and perform MSBL-STAP, which is called ISV-MSBL.

### 4.1. Gain and Phase Errors

In this experiment, we verify the performance of the proposed two-stage SR-STAP method in the presence of gain and phase errors. $G_{gain}=diag\left(\left[g_{1},g_{2},\cdots ,g_{N}\right]\right)$ is the error matrix, $g_{1}\;=\;1$, $g_{i}=\left(1+\Delta \alpha_{i}\right)e^{j\Delta \varphi_{i}}\left(i=2,\cdots ,N\right)$ is the gain and phase error of the *i*th element, where $\Delta \alpha_{i}$ and $\Delta \varphi_{i}$ follow a uniform distribution within $\left[-0.1,0.1\right]$ and $\left[-10^{\circ },10^{\circ }\right]$, respectively [47]. Then, we can get the following equation

(50) $$\hat{A}={\hat{G}}_{gain}A$$

where $\hat{A}\;=\;\left[\hat{a}\left(f_{s1}^{}\right),\hat{a}\left(f_{s2}^{}\right),\ldots ,\hat{a}\left(f_{sK_{1}}^{}\right)\right]$ is the matrix whose columns are the $K_{1}$ estimated spatial steering vectors with array errors in the first stage, $A\;=\;\left[\bar{a}\left(f_{s1}^{}\right),\bar{a}\left(f_{s2}^{}\right),\ldots ,\bar{a}\left(f_{sK_{1}}^{}\right)\right]$ is the matrix whose columns are the $K_{1}$ ideal spatial steering vectors without array errors, ${\hat{G}}_{gain}$ is the estimate of $G_{gain}$. The least square (LS) solution of ${\hat{G}}_{gain}$ is given by

(51) $${\hat{G}}_{gain}\;=\;\hat{A}A^{H}{\left(AA^{H}\right)}^{-1}$$

**Figure 4:**
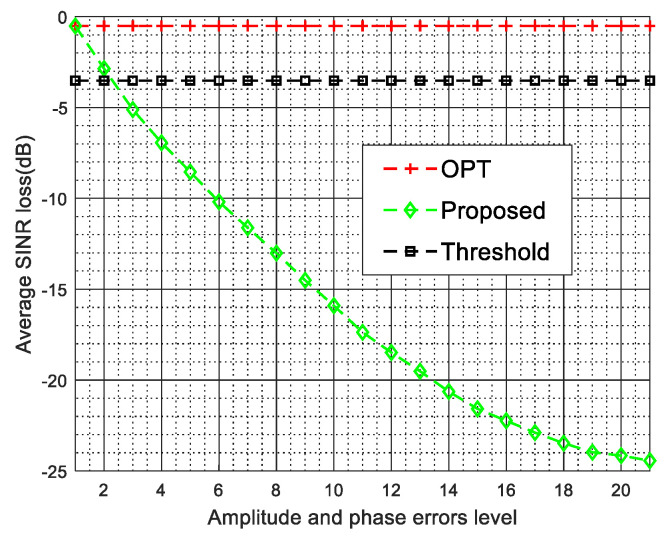
Average SINR loss versus the amplitude and phase errors level.

To show the performance loss of the proposed method where there are varying levels of amplitude and phase errors, twenty-one different levels of the amplitude and phase errors are defined as “level1”, “level2”, “level3”, ⋯, “level20” and “level21”, which are subject to uniform distribution as (0,0)/(0${}^{\circ }$,0${}^{\circ }$), (−0.01,0.01)/(−1${}^{\circ }$,1${}^{\circ }$), (−0.02,0.02)/(−2${}^{\circ }$,2${}^{\circ }$), ⋯, (−0.19,0.19)/(−19${}^{\circ }$,19${}^{\circ }$) and (−0.2,0.2)/(−20${}^{\circ }$,20${}^{\circ }$), respectively. Figure 4 plots the average SINR loss versus the amplitude and phase errors level, as shown in Figure 4, the higher the amplitude and phase errors level, the severer performance loss of the proposed method. In general, it is acceptable when the performance loss of the algorithm is less than 3 dB. In Figure 4, the black dotted line with a square mark denotes a threshold value, which means that the average SINR loss is decreased by 3 dB compared with the OPT. From Figure 4, we can observe that the slight amplitude and phase errors will cause a severe performance loss of the proposed method, specifically, the performance loss of the proposed two-stage method is greater than 3 dB when the amplitude and phase errors level is greater than 2. Thus, we can say that when the phase errors level is greater than 2, the performance of the proposed method is significantly deteriorated.

The gain and phase errors estimated by the proposed method are presented in Table 2, from this table, it is observed that the estimated values are very close to the true ones.

**Figure 5:**
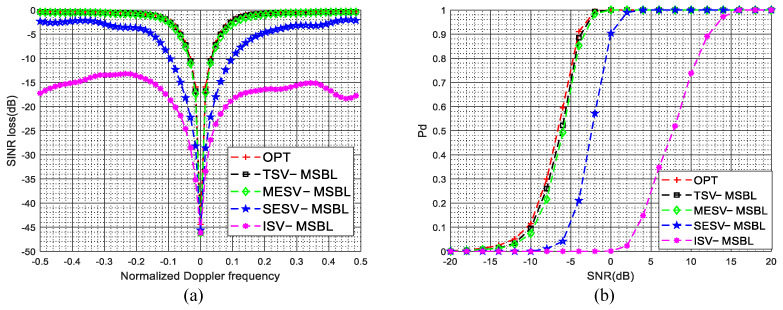
SINR loss curves and PD versus SNR curves in the presence of gain and phase errors. (**a**) SINR loss versus the normalized Doppler frequency in the presence of gain and phase errors; (**b**) PD versus SNR ($f_{st}\;=\;0.1$) in the presence of gain and phase errors.

The SINR loss curves in the presence of gain and phase errors are given in Figure 5a. As shown in Figure 5a, due to the steering vector mismatch between the dictionary and clutter data, the clutter suppression performance of the ISV-MSBL method is much poorer than that of the TSV-MSBL method. By comparing the SINR loss curves of ISV-MSBL, SESV-MSBL, MESV-MSBL and the OPT, it is observed that the MESV-MSBL method achieves the comparable performance as the OPT, which is better than that of the SESV-MSBL method and much better than that of the ISV-MSBL method. The results demonstrate that the gain and phase errors can be well calibrated by the developed steering vector estimation method. In the sidelobe region ($f_{d}^{}\;=\;0.4$), compared with the ISV-MSBL method, the output SINRs of proposed SESV-MSBL method and MESV-MSBL method are increased by about 14.1 dB and 16.3 dB, respectively. In the mainlobe region ($f_{d}^{}\;=\;0.1$), compared with the ISV-MSBL method, the output SINRs of proposed SESV-MSBL method and MESV-MSBL method are increased by about 8.5 dB and 15.8 dB, respectively. Whether in the mainlobe region or in the sidelobe region, the clutter suppression performance of the proposed method is significantly improved. The PD versus SNR curves in the presence of gain and phase errors are given in Figure 5b. As depicted in Figure 5b, the target detection performance of the MESV-MSBL method is close to the optimal performance, which is better than that of the SESV-MSBL method and much better than that of the ISV-MSBL method. Compared with the ISV-MSBL method, the slow-moving target detection performance of proposed SESV-MSBL method and MESV-MSBL method are significantly improved.

### 4.2. Mutual Coupling

In this experiment, we verify the performance of the proposed two-stage SR-STAP method in the presence of mutual coupling. Assuming that mutual coupling coefficient can be ignored when the element spacing is greater than 1.5 wavelength, which means that q=3. We set the non-zero mutual coupling coefficients as 1, 0.1250+0.2165j, 0.0866−0.0500j, respectively [49]. The same principle as the estimation of $G_{gain}$, we can also get the estimate of mutual coupling matrix $G_{mutual}$ by Equation (51).

The mutual coupling coefficients estimated by the proposed method are presented in Table 3, from this table, we find that the estimated values are also very close to the true ones.

**Figure 6:**
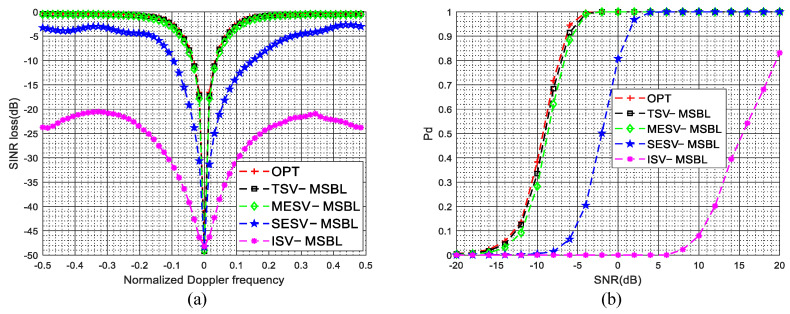
SINR loss curves and PD versus SNR curves in the presence of mutual coupling. (**a**) SINR loss versus the normalized Doppler frequency in the presence of mutual coupling; (**b**) PD versus SNR ($f_{st}\;=\;0.1$) in the presence of mutual coupling.

The SINR loss curves in the presence of mutual coupling are depicted in Figure 6a, the PD versus SNR curves in the presence of mutual coupling are depicted in Figure 6b. The superior clutter suppression performance and target detection performance of the proposed method are demonstrated. In the sidelobe region ($f_{d}^{}\;=\;0.4$), compared with the ISV-MSBL method, the output SINRs of proposed SESV-MSBL method and MESV-MSBL method are increased by about 19.3 dB and 21.7 dB, respectively. In the mainlobe region ($f_{d}^{}\;=\;0.1$), compared with the ISV-MSBL method, the output SINRs of proposed SESV-MSBL method and MESV-MSBL method are increased by about 17.2 dB and 27.6 dB, respectively.

### 4.3. Sensor Location Errors

In this experiment, we verify the performance of the proposed two-stage SR-STAP method in the presence of sensor location errors. $\Delta_{\mathrm{PE}}=diag\left(\left[\Delta_{0},\Delta_{1},\ldots ,\Delta_{N-1}\right]\right)$ is the position errors matrix, $\Delta_{0}\;=\;0$, $\Delta_{i-1}\left(i=2,\cdots ,N\right)$ is the position error value of the *i*th element, where $\Delta_{i-1}$ follows a uniform distribution within $\left[-0.1d,0.1d\right]$ [52]. We can also utilize the $K_{1}$ estimated spatial steering vectors $\left[\hat{a}\left(f_{s1}^{}\right),\hat{a}\left(f_{s2}^{}\right),\ldots ,\hat{a}\left(f_{sK_{1}}^{}\right)\right]$ and the $K_{1}$ ideal spatial steering vectors $\left[\bar{a}\left(f_{s1}^{}\right),\bar{a}\left(f_{s2}^{}\right),\ldots ,\bar{a}\left(f_{sK_{1}}^{}\right)\right]$ to estimate $\Delta_{i-1}$, given by

(52) Δ^i−1=1K1∑k=1K1λanglea^ifska^ifska¯ifska¯ifsk2πcosϕk

where ${\hat{a}}_{i}\left(f_{sk}^{}\right)$ and ${\bar{a}}_{i}\left(f_{sk}^{}\right)$ are the *i*th element of $\hat{a}\left(f_{sk}^{}\right)$ and $\bar{a}\left(f_{sk}^{}\right)$, respectively. $\phi_{k}$ is the is the spatial cone angle corresponding to the center frequency of the *k*th Doppler filter.

The sensor location errors estimated by the proposed method are presented in Table 4, from this table, we find that the estimated ones match true ones pretty well.

**Figure 7:**
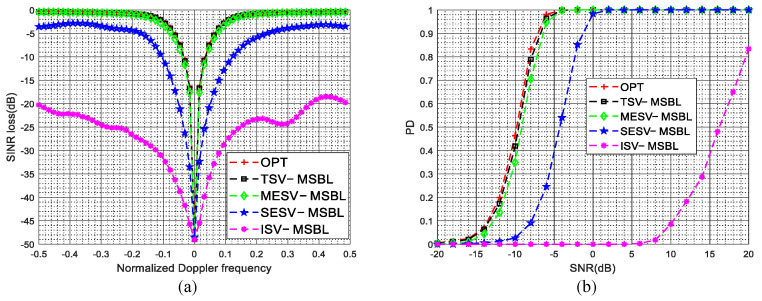
SINR loss curves and PD versus SNR curves in the presence of sensor location errors. (**a**) SINR loss versus the normalized Doppler frequency in the presence of sensor location errors; (**b**) PD versus SNR ($f_{st}\;=\;0.1$) in the presence of sensor location errors.

The SINR loss curves in the presence of sensor location errors are depicted in Figure 7a, the PD versus SNR curves in the presence of sensor location errors are depicted in Figure 7b. It is observed that the clutter suppression performance and the target detection performance of the proposed method are significantly improved when sensor location errors are present. In the sidelobe region ($f_{st}\;=\;0.4$), compared with the ISV-MSBL method, the output SINRs of proposed SESV-MSBL method and MESV-MSBL method are increased by about 15.5 dB and 18.2 dB, respectively. In the mainlobe region ($f_{st}\;=\;0.1$), compared with the ISV-MSBL method, the output SINRs of proposed SESV-MSBL method and MESV-MSBL method are increased by about 15.9 dB and 26.2 dB, respectively.

### 4.4. Arbtrary Array Errors

In this experiment, we model the arbitrary array errors as the combined effects of gain and phase errors, mutual coupling and sensor location errors, then, the performance of proposed method in the presence of arbitrary array errors is demonstrated. The specific values of array errors are the same as those in Section 4.1, Section 4.2 and Section 4.3.

**Figure 8:**
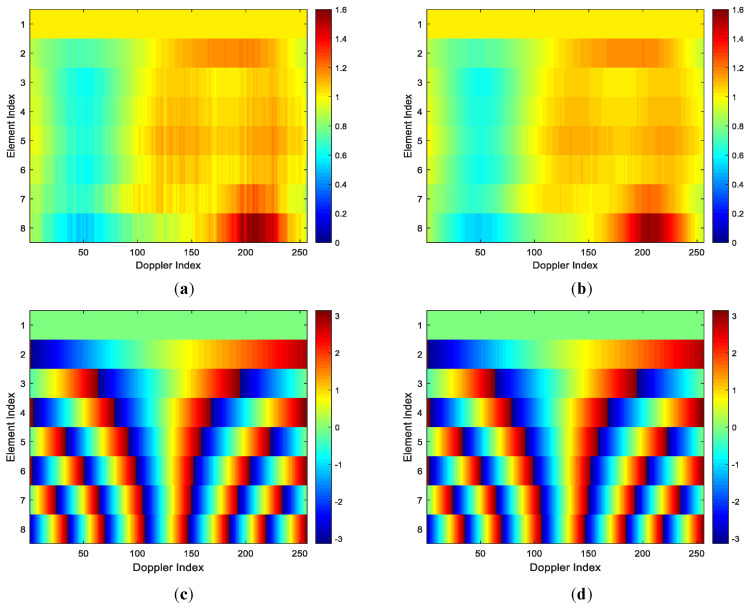
Steering vector estimation results of all Doppler bins. (**a**) Amplitudes of estimated steering vectors; (**b**) Amplitudes of true steering vectors; (**c**) Interferometry phases of estimated steering vectors; (**d**) Interferometry phases of true steering vectors.

The amplitudes and interferometry phases of all $K_{1}$ estimated spatial steering vectors with array errors in the first stage are given in Figure 8a,c, respectively. The amplitudes and interferometry phases of $K_{1}$ true spatial steering vectors with array errors are given in Figure 8b,d, respectively. From Figure 8a,c, we can observe that the amplitudes of the estimated spatial steering vectors with array errors are close to that of the true spatial steering vectors. From Figure 8b,d, we can also observe that the interferometry phases of the estimated spatial steering vectors with array errors are very close to that of the true spatial steering vectors. Thus, we can say that the amplitudes differences and the phase differences between the estimated spatial steering vectors and true spatial steering vectors of all Doppler bins are very small, i.e., a set of $K_{1}$ spatial steering vectors can be well estimated in the first stage of our two-stage SR-STAP method in the presence of arbitrary array errors.

**Figure 9:**
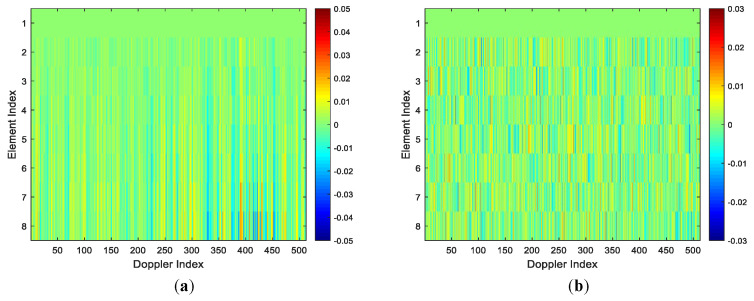
The amplitude differences and phase differences between the estimated spatial steering vectors and true spatial steering vectors (all Doppler bins). (**a**) Amplitude differences; (**b**) Phase differences.

**Figure 10:**
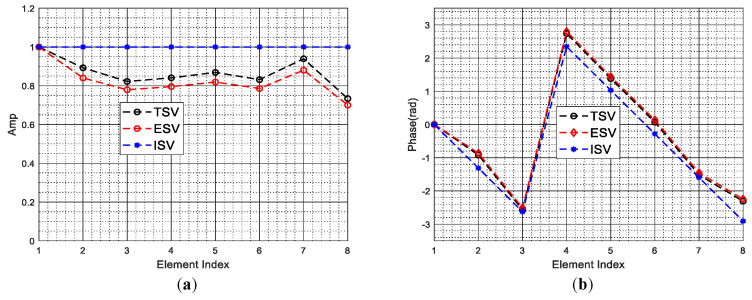
Performance comparison on steering vector estimation. (**a**) Amplitude; (**b**) Phase. TSV denotes the true steering vector with array errors, ESV denotes the estimated steering vector with array errors, and ISV denotes the ideal steering vector without array errors.

Figure 9a,b show the amplitudes differences and the phase differences between the estimated spatial steering vectors and true spatial steering vectors of all Doppler bins, respectively. As depicted in Figure 9, the amplitude differences and the phase differences between the estimated spatial steering vectors and true spatial steering vectors of all Doppler bins are very small, i.e., the estimated spatial steering vectors are very close to these true spatial steering vectors. Thus, when the arbitrary array errors are present, a set of spatial steering vectors can be well estimated in the first stage of our two-stage method. The results intuitively demonstrate the superior steering vector estimation performance of the proposed method. For clarify, in Figure 10, the amplitudes and phases of the ideal steering vector, the true steering vector and the estimated steering vector of the 75th Doppler bin are demonstrated, the results indicate that the estimated steering vector is much closer to the true steering vector than the ideal steer vector, and the true steering vector can be well approximated by the estimated steering vector in the presence of arbitrary array errors.

**Figure 11:**
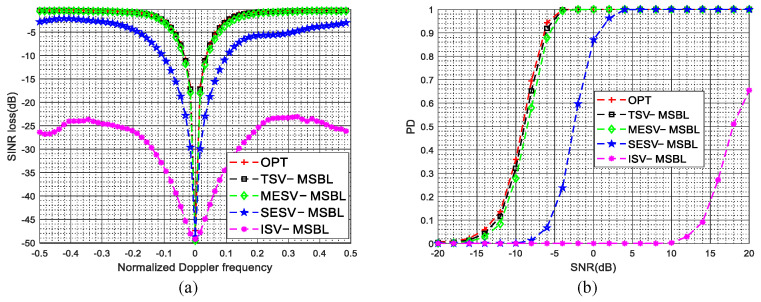
SINR loss curves and PD versus SNR curves in the presence of arbitrary array errors. (**a**) SINR loss versus the normalized Doppler frequency in the presence of arbitrary array errors; (**b**) PD versus SNR ($f_{st}\;=\;0.1$) in the presence of arbitrary array errors.

The SINR loss curves and the PD versus SNR curves in the presence of arbitrary array errors are depicted in Figure 11a,b, respectively. From Figure 11, it is observed that the ISV-MSBL method has a severe performance degradation when arbitrary array errors are present. However, compared with the ISV-MSBL method, the clutter suppression performance and the target detection performance of the proposed SESV-MSBL method and MESV-MSBL method are significantly improved and the MESV-MSBL method can obtain the comparable performance as the OPT. The reason is that the array errors are well calibrated by the developed steering vector estimation method and thereby the mismath problem between the clutter data and the space-time dictionary are well solved. The results further validate the superior performance of the proposed method. In the sidelobe region ($f_{d}^{}\;=\;0.4$), compared with the ISV-MSBL method, the output SINRs of proposed SESV-MSBL method and MESV-MSBL method are increased by about 20.5 dB and 23.8 dB, respectively. In the mainlobe region ($f_{d}^{}\;=\;0.1$), compared with the ISV-MSBL method, the output SINRs of proposed SESV-MSBL method and MESV-MSBL method are increased by about 23.6 dB and 30.7 dB, respectively.

**Figure 12:**
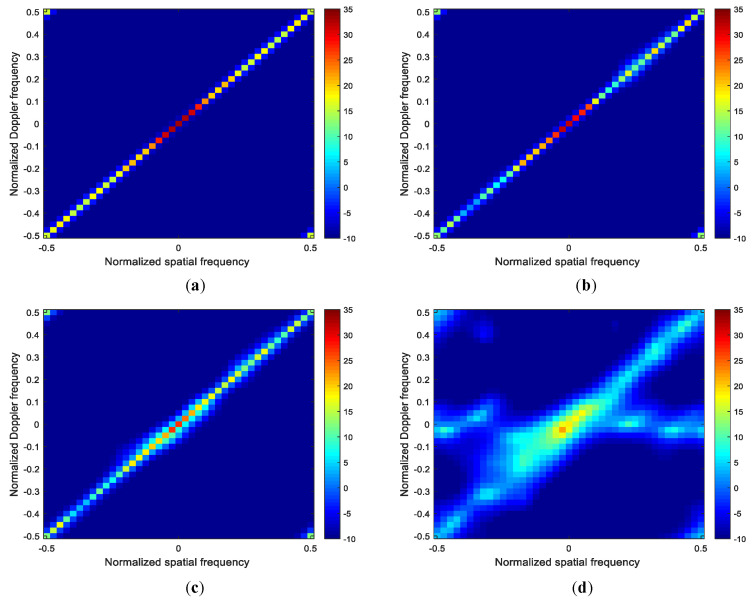
Clutter capon spectra of different methods. (**a**) OPT-STAP; (**b**) MESV-MSBL; (**c**) SESV-MSBL; (**d**) ISV-MSBL.

To better illustrate the advantage of the proposed method, Figure 12a–d plot the clutter capon spectra of different STAP methods. From Figure 12a–c, we can observe that the spectra of the MESV-MSBL method is the closest to the optimal spectra with few clutter power leakage, and the spectra of the SEMV-MSBL method is close to the optimal spectra with some clutter power leakage and a slight spectrum expansion. However, from Figure 12d, we can observed that the spectra of the ISV-MSBL method has severe clutter power leakage and spectrum expansion, the reason is that if the array calibration is not performed, the steering vector mismatch between the clutter data and the space-time dictionary will cause that the clutter spectrum cannot be well estimated; thus, the clutter suppression performance and the slow moving target detection performance of the SR-STAP methods will significantly degrade for the reason that the adaptive pattern cannot to suppress clutter and protect the target well because of the widened notches or the incorrect notches. That is the reason why we must perform array calibration when we apply sparse recovery technique to STAP.

**Figure 13:**
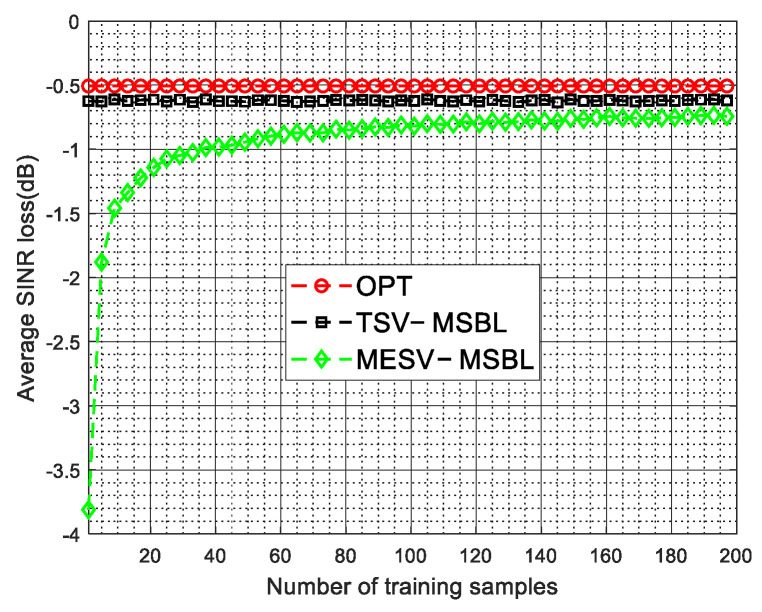
Average SINR loss versus the number of training samples used in the first stage.

Figure 13 plots the average SINR loss versus the number of training samples used in the first stage. From Figure 13, we can know that when the number of training samples used in the first stage is larger than 100, the spatial steering vectors with array errors can be well estimated and the MESV-MSBL method can acquire comparable performance as the TSV-MSBL method and the OPT.

**Figure 14:**
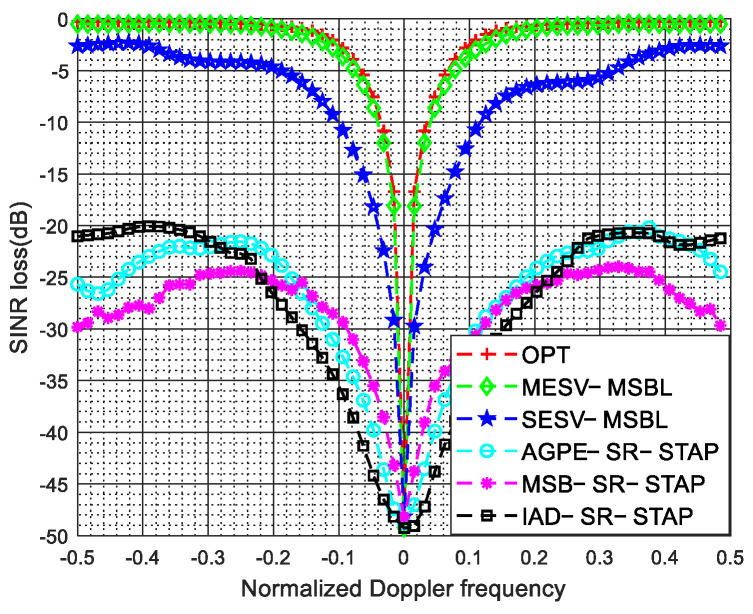
SINR loss comparison of different methods in the presence of arbitrary array errors.

In Figure 14, we compare the clutter suppression performance of the proposed SESV-MSBL method and the MESV-MSBL method with that of the AGPE-SR-STAP method [47], the MSB-SR-STAP method [49] and the IAD-SR-STAP method [48]. From Figure 14, we can observe that the SESV-MSBL method and the MESV-MSBL method have narrower notches than other STAP methods. The reason is that AGPE-SR-STAP method and IAD-SR-STAP method are only suitable for gain/phase calibration and MSB-SR-STAP method is only suitable for mutual coupling calibration. Thus, in the presence of arbitrary array errors, these methods are not effective any more.

**Figure 15:**
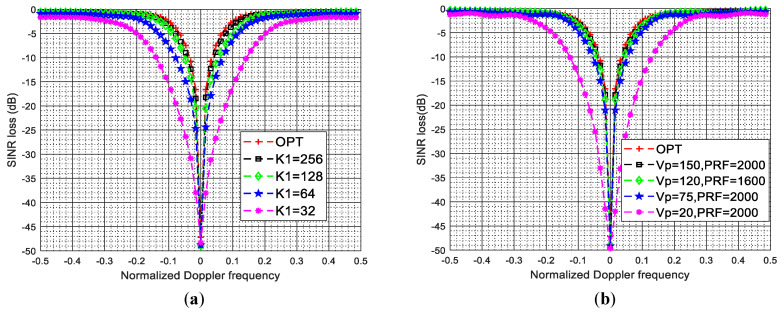
SINR loss curves of the MESV-MSBL method under different system parameters. (**a**) SINR loss curves of the MESV-MSBL method under different pulse numbers in a CPI in the first stage of the proposed two-stage SR-STAP method; (**b**) SINR loss curves of the MESV-MSBL method under different platform velocities and pulse repetition frequencies.

Finally, we give two experiments to show that how much variation in values of the system parameters in Table 1 affect the performance of the proposed method. Figure 15a plots the SINR loss curves of the MESV-MSBL method under different pulse numbers in a CPI in the first stage of the proposed two-stage SR-STAP method. From Figure 15a, we can observe that the more pulses in a CPI, the better clutter suppression performance of the proposed MESV-MSBL method. The reason is that when the number of pulses $K_{1}$ in a CPI increases, the sharpening ratio κ given in (20) will become larger and the width of the spatial frequency passband Δ given in (19) will become smaller, as a result, the correlation coefficient of $a\left(f_{si}^{}\right)$ and $a\left(f_{si}\;+\;\Delta /2\right)$ given in (21) will become larger. In other word, as the number of pulses $K_{1}$ increases, the spatial steering vectors can be estimated more and more accurately in the first stage of our two-stage SR-STAP method, as a result, the clutter suppression performance of the proposed MESV-MSBL method is getting better and better. Thus, we can conclude that the system parameters determine the value of the sharpening ratio κ and the value of the sharpening ratio κ determines the clutter suppression performance of the proposed two-stage SR-STAP method. The greater the sharpening ratio κ, the better the clutter suppression performance of the proposed method. In general, as long as these system parameters can guarantee that the correlation coefficient of $a\left(f_{si}^{}\right)$ and $a\left(f_{si}\;+\;\Delta /2\right)$ given in (21) is greater than 0.95, the proposed two-stage SR-STAP method can obtain superior clutter suppression performance. To further confirm our conclusion, Figure 15b plots the SINR loss curves of the MESV-MSBL method under different platform velocities and pulse repetition frequencies. From Figure 15b, we can observe that when $v_{p}=150$ and $f_{PRF}=2000$, the proposed MESV-MSBL method can achieve superior clutter suppression performance for the reason that the sharpening ratio κ is high in this case. In addition, when $v_{p}=120$ and $f_{PRF}=1600$, although the system parameters have changed, the sharpening ratio κ has not changed; thus, the proposed MESV-MSBL method can still achieve superior clutter suppression performance. However, when $v_{p}=120$, $f_{PRF}=2000$ and $v_{p}=20$, $f_{PRF}=2000$, the clutter suppression performance of the proposed MESV-MSBL method is getting worse and worse because that the sharpening ratio κ becomes smaller and smaller.

### 4.5. Arbitrary Array Errors and Intrinsic Clutter Motion

In this experiment, we verify the performance of the proposed two-stage SR-STAP method in the presence of arbitrary array errors and ICM. The same to Section 4.4, we model the arbitrary array errors as the combined effects of gain and phase errors, mutual coupling and sensor location errors. In this experiment, we only consider the ICM in the first stage of our two-stage method, i.e., we only measure the effect of the presence of ICM on estimating the spatial steering vectors in the first stage, without considering the clutter spectrum expansion problem caused by ICM in the second stage. In fact, this problem can be effectively handled by the covariance matrix taper (CMT) approach, the interested reader is referred to the literature [53,54] for further details. The ICM model is given by [1]. The temporal autocorrelation of the fluctuations is Gaussian in shape

(53) $$\gamma \left(k\right)=\exp\left\{-\frac{8\pi^{2}\sigma_{v}^{2}T_{r}^{2}}{\lambda^{2}}k^{2}\right\}$$

where $\sigma_{v}$ is the velocity standard deviation, Tr=11fPRFfPRF is the pulse repetition interval.

**Figure 16:**
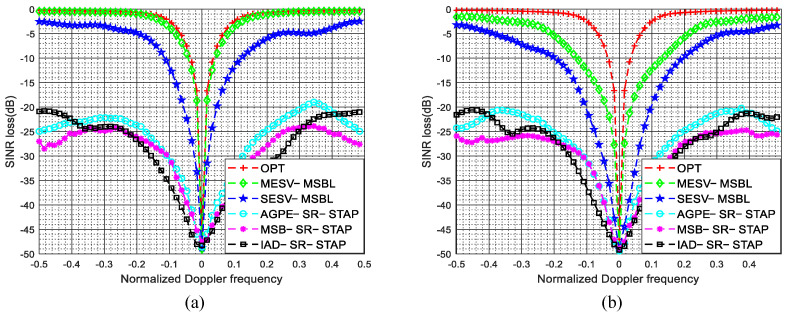
SINR loss comparison of different methods in the presence of arbitrary array errors and intrinsic clutter motion. (**a**) σv=0.5mmss; (**b**) σv=3mmss.

The SINR loss curves of different methods in the presence of arbitrary array errors and ICM are depicted in Figure 16. As shown in Figure 16a, when σv=0.5mmss, due to the broadening of the Doppler spectrum caused by ICM is much smaller than the DFPW of the heavy tapered Doppler filter, the proposed two-stage method still achieves superior performance. Specifically, in this experiment, when σv=0.5mmss, the width of the Doppler spectrum is Db=2σv=2σvλfPRFλfPRF=1=2σv=2σvλfPRFλfPRF=1300300, and a reasonable DFPW value is 55K1K1, i.e., Dw=5Dw=5256256; thus, the inequality $D_{b}\ll D_{w}$ holds and we can say that the broadening of the Doppler spectrum has little effect on estimating the spatial steering vectors. However, when σv=3mmss, the width of the Doppler spectrum is Db=2σvDb=2σvλfPRFλfPRF=6Db=2σvDb=2σvλfPRFλfPRF=6300300 and the inequality $D_{b}\ll D_{w}$ no longer holds; thus, the broadening of the Doppler spectrum will have some adverse effect on estimating spatial steering vectors. As depicted in Figure 16b, when σv=3mmss, due to severe temporal fluctuations, the notches of the proposed SESV-MSBL method and MESV-MSBL method are spreading. However, compared with other SR-STAP methods, the proposed two-stage method still achieves better performance.

**Figure 17:**
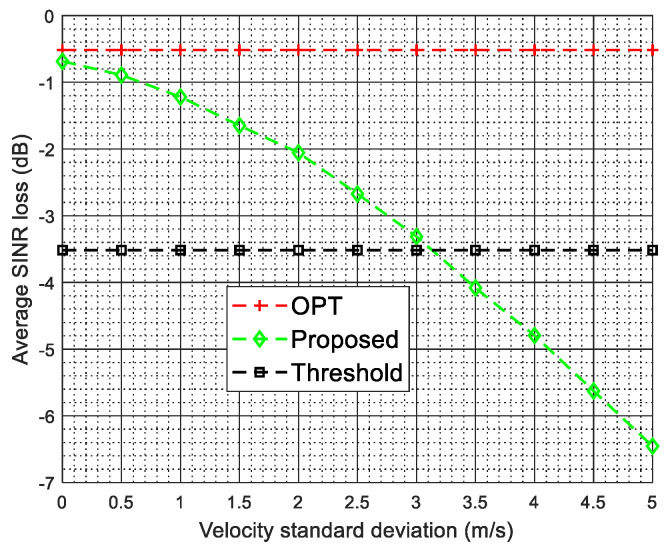
Average SINR loss versus the velocity standard deviation.

The existence of intrinsic clutter motion will deteriorate the performance of the proposed two-stage SR-STAP method. The more serious the intrinsic clutter motion, the less accurate the estimation of the spatial steering vectors at the first stage our two-stage method, thereby the worse the algorithm performance. In general, it is acceptable when the performance loss of the algorithm is less than 3 dB. Figure 17 plots the average SINR loss versus the velocity standard deviation. As shown in Figure 17, when the velocity standard deviation is small, the proposed two-stage method still obtains the near-optimal performance, however, when the velocity standard deviation becomes larger, the performance of the proposed method degrades due to the severer pulse-to-pulse fluctuations. In Figure 17, the black dotted line with a square mark denotes a threshold value, which means that the average SINR loss is decreased by 3 dB compared with the OPT. From Figure 17, we can observe that the performance loss of the proposed two-stage method is less than 3dB when the velocity standard deviation is less than 3.1 m/s. For land clutter, in some areas, such as rural and urban, its velocity standard deviation is usually a very small value, even in wooded terrain, its velocity standard deviation is generally less than 1mmss [55]. From Figure 17, we can observe that the performance loss of the proposed two-stage method is less than 1 dB when the velocity standard deviation is less than 1 m/s. Therefore, we can say that the performance of the proposed two-stage method is satisfactory when the velocity standard deviation is less than 1 m/s. Thus, the proposed method is still effective for ground clutter suppression and ground moving target detection in the presence of arbitrary array errors and small ICM.

## 5. Conclusions

The model mismatch caused by array errors drastically degrade the clutter suppression performance and the target detection performance of SR-STAP methods. To solve this problem, a new two-stage SR-STAP method is proposed in this paper. In our two-stage SR-STAP method, firstly, based on the spatial-temporal coupling property of ground clutter data, we obtain a set of spatial steering vectors with array errors by fine Doppler localization, then, in order to solve the model mismatch problem caused by array errors, we directly use these obtained spatial steering vectors with array errors to construct the space-time dictionary, finally, the constructed space-time dictionary and MSBL algorithm are combined for space-time adaptive processing. The simulation results demonstrate that the variation in system parameters will affect the performance of the proposed two-stage SR-STAP method, the system parameters determine the value of the sharpening ratio κ and the value of the sharpening ratio κ determines the performance of the proposed two-stage SR-STAP method. The greater the sharpening ratio κ, the better the clutter suppression performance and the target detection performance of the proposed method. In general, as long as these system parameters can guarantee that the correlation coefficient of $a\left(f_{si}^{}\right)$ and $a\left(f_{si}\;+\;\Delta /2\right)$ given in (21) is greater than 0.95, the proposed two-stage SR-STAP method can obtain favorable performance. In addition, this simulation results which are obtained based on some reasonable system parameters which are listed in Table 1 demonstrate that the spatial steering vectors with array errors can be well estimated in the first stage of our two-stage SR-STAP method when the arbitrary array errors and small ICM are present, and also demonstrate that the proposed method can achieve superior clutter suppression performance and target detection performance in the presence of arbitrary array errors.

## Figures and Tables

**Table 1:** Radar system parameters.

Parameter	Value
Bandwidth	2.5 M
Wavelength	0.3 m
Pulse repetition frequency	2000 Hz
Platform velocity	150 m/s
Platform height	9 km
Element number	8
Pulse number in the first stage	256
Pulse number in the second stage	8
CNR	40 dB

**Table 2:** Gain and phase errors estimation.

	True	Estimated
g1	1	1
g2	0.9178 + j0.0642	0.9183 + j0.0644
g3	1.1288 + j0.0461	1.1298 + j0.0466
g4	0.8951 + j0.0941	0.8965 + j0.0944
g5	0.9277 + j0.0649	0.9291 + j0.0651
g6	0.8888 + j0.0466	0.8898 + j0.0469
g7	1.0946 + j0.0951	1.0955 + j0.0956
g8	0.8988 + j0.0471	0.8985 + j0.0471

**Table 3:** Mutual coupling estimation.

	True	Estimated
c1	1	1
c2	0.1250 + j0.2165	0.1253 + j0.2169
c3	0.0866 + j0.0500	0.0869 + j0.0498

**Table 4:** Sensor location errors estimation.

	True (m)	Estimated (m)
$\Delta_{0}$	0	0
$\Delta_{1}$	−0.0041	−0.0042
$\Delta_{2}$	0.004	0.0041
$\Delta_{3}$	0.0003	0.0002
$\Delta_{4}$	−0.014	−0.0139
$\Delta_{5}$	0.0123	0.0122
$\Delta_{6}$	−0.0021	−0.0022
$\Delta_{7}$	−0.0135	−0.0135
